# Use of endovascular embolization to treat a ruptured arteriovenous malformation in a pregnant woman: a case report

**DOI:** 10.1186/1752-1947-6-113

**Published:** 2012-04-23

**Authors:** Walter J Jermakowicz, Luke D Tomycz, Mayshan Ghiassi, Robert J Singer

**Affiliations:** 1 Department of Neurological Surgery, Vanderbilt University Medical Center, T-4224 Medical Center North, Nashville, Tennessee 37232-2380, USA

## Abstract

**Introduction:**

Pregnancy has been linked to increased rates of arteriovenous malformation rupture. This link remains a matter of debate and very few studies have addressed the management of arteriovenous malformation in pregnancy. Unruptured arteriovenous malformations in pregnant woman generally warrant conservative management due to the low rupture risk. When pregnant women present with ruptured arteriovenous malformation, however, surgery is often indicated due to the increased risk of re-rupture and associated mortality. Endovascular embolization is widely accepted as an important component of contemporary, multimodal therapy for arteriovenous malformations. Although rarely curative, embolization can facilitate subsequent surgical resection or radiosurgery. No previous reports have been devoted to the endovascular management of an arteriovenous malformation in a pregnant woman.

**Case presentation:**

A 23-year-old Caucasian woman presented with headache and visual disturbance after the rupture of a left parieto-occipital arteriovenous malformation in the 22nd week of her pregnancy. After involving high-risk obstetric consultants and taking precautions to shield the fetus from ionizing radiation, we proceeded with a single stage of endovascular embolization followed soon after by open surgical resection of the arteriovenous malformation. There were several goals for the angiography in this patient: to better understand the anatomy of the arteriovenous malformation, including the number and orientation of feeding arteries and draining veins; to look for associated pre-nidal or intra-nidal aneurysms; and to partially embolize the arteriovenous malformation via safely-accessible feeders to facilitate surgical resection and minimize blood loss and operative morbidity.

**Conclusion:**

From our experience and review of the literature, we maintain that ruptured arteriovenous malformations in pregnancy may be managed in a similar manner to those in non-gravid women. Precautions should be taken to reduce the operative time and exposure of the fetus to ionizing radiation and contrast agents.

## Introduction

The rupture of an intracranial arteriovenous malformation (AVM) in pregnancy is a rare occurrence, but may have fatal consequences [1]. A link between AVM rupture and pregnancy has been proposed; it may be caused by the increased cardiac output or circulatory effects of the elevated estrogen levels [2]. However, in some series the reported hemorrhage rate from AVMs in pregnancy is around 0.6% to 3.5%, which is similar to the 2% to 4% rate in non-pregnant women. Due to this low risk of hemorrhage, most authors recommend conservative management of unruptured AVMs during pregnancy [2,3]. An aggressive approach is warranted, however, when pregnant patients present with a ruptured AVM. The risk of re-bleed during the same pregnancy (27% to 30%) is greater than the risk of re-bleed in non-gravid women within one year of their initial bleed (6%). These bleeds in pregnant women are associated with high maternal and fetal mortality (10% to 40%) [2,4]. Thus, despite the potentially deleterious effects of radiation on the fetus, surgical management is generally indicated when pregnant women present with a ruptured AVM.

Historically, ruptured AVMs in pregnant women have been managed surgically with great success [2,3,5]. In patients presenting with AVM rupture, resection has been shown to be associated with lower rates of maternal and fetal mortality when compared to conservative management [4,6]. More recently, with advances in endovascular technology, embolization of AVMs has been used as an adjuvant to surgery, if not as a primary mode of treatment, with much success. However, the role of endovascular embolization in the management of AVM rupture in pregnant women has not been established.

Here, we report the case of a 22-week-pregnant woman who presented with a symptomatic ruptured AVM, treated with embolization followed by surgical resection. There were several goals for the angiography in this patient: to better understand the anatomy of the AVM, including the number and orientation of feeding arteries and draining veins; to look for associated pre-nidal or intra-nidal aneurysms; and to partially embolize the AVM via safely-accessible feeders to facilitate surgical resection and minimize blood loss and operative morbidity.

## Case presentation

**Figure 1:**
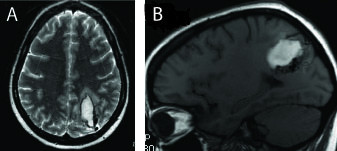
**Magnetic resonance imaging of the left parieto-occipital arteriovenous malformation**. T2-weighted magnetic resonance imaging scans of the malformation and associated hematoma from the outside hospital showing **(A) **axial and **(B) **sagittal views.

A 23-year-old Caucasian woman, 22 weeks into her first pregnancy, presented to an outside hospital following the sudden onset of severe headache associated with nausea and vomiting. She denied weakness or numbness, but reported occasional double vision. Her past medical history was significant for an irregular heartbeat, gastroesophageal reflux disease and hypertension. Our patient's only physical exam finding was a right lower visual field deficit but otherwise she was intact. Non-contrasted computed tomography of her head revealed an acute left parieto-occipital hemorrhage with mild mass effect. Magnetic resonance imaging (MRI) revealed a 5 × 2.6 cm hematoma in the left parieto-occipital region abutting the ventricle with flow voids, characteristic of an AVM (Figure 1). Mild mass effect was noted with no midline shift or effacement of basilar cisterns. Digital subtraction cerebral angiography demonstrated that the nidus was being supplied by the posterior branches of her left middle cerebral, left anterior and posterior cerebral arteries. The majority of the venous drainage was via her superior sagittal sinus. No intra-nidal or flow-related aneurysms were noted. Our patient was counseled with regards to her options and opted for endovascular embolization followed by surgical resection of the AVM.

**Figure 2:**
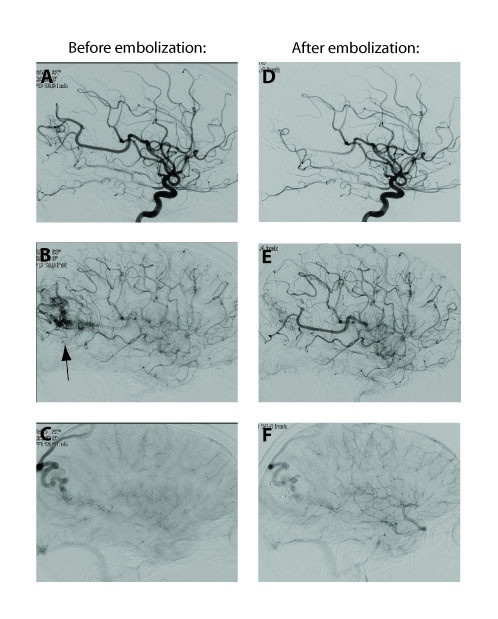
**Sagittal angiography comparing the vasculature after injection of the contrast agent through the left middle cerebral pedicle, before and after endovascular embolization**. **(A and D) **show the early arterial phase. **(B and E) **show the late arterial phase. The black arrow in B shows the arteriovenous malformation nidus. **(C and F) **show images during the capillary phase. The large tortuous draining vein is clearly visible in place of the arteriovenous malformation nidus. We estimated a 50% reduction in arteriovenous malformation size after embolization.

Our patient was brought to the endovascular suite three weeks after her initial hemorrhage. The procedure was coordinated with obstetricians as well as anesthesiologists to ensure the safety of both the mother and fetus. During the procedure, various measures were taken to minimize exposure of the fetus to ionizing radiation. Our patient's abdomen was fully covered with lead drapes. In addition, instead of performing a full angiogram prior to the embolization, we used a prior MRI scan to determine the primary pedicle with the greatest promise of having nidal feeders and then selectively targeted that vessel during the embolization procedure. The guide catheter was positioned in her distal left internal carotid artery. A microcatheter was navigated over a guidewire to select the inferior division of the middle cerebral artery feeding the AVM. Visipaque™ 320 (iodixanol) contrast agent was used at half strength. After ensuring the selected branch was feeding the nidus, Onyx-18 (Onyx Liquid Embolic System^®^) was used in the standard fashion [7-9] to embolize the AVM. Control runs after embolization revealed the successful reduction of the nidus by approximately 50%, preserving venous outflow (Figure 2). After the procedure, our patient was transferred to the neurosurgical intensive care unit for close observation. She had an uncomplicated postoperative course.

Three weeks later, our patient was brought to the neurosurgical operating suite and anesthetized by obstetric anesthesia. The obstetrics team was also in the room and was monitoring the fetus during the entire procedure. After insertion of a right femoral sheath in the standard fashion, our patient was put in the prone position for the craniotomy. Stealth guidance was used to plan our craniotomy prior to incision. The craniotomy was centered over the left parieto-occipital AVM and using the standard microsurgical technique we proceeded to safely and effectively resect the AVM. Once we were satisfied with our resection we performed an intraoperative cerebral angiogram. Once again, all possible measures were taken to reduce radiation and contrast exposure to mother and fetus. The angiogram revealed complete resection of the AVM. The wound was closed in the standard fashion and our patient was transferred to the neurosurgical intensive care unit.

Our patient recovered well without complications and was discharged home on postoperative day two. At the time of this submission, our patient had delivered her baby at 39 weeks via a normal vaginal delivery at an outside hospital without the need for high-risk obstetric personnel. Both mother and baby are healthy and with no complications related to the procedures.

## Discussion

Given the increased risk of re-hemorrhage in pregnant mothers that present with AVM bleed and the high associated maternal and fetal mortality [2,6,10], aggressive management of these lesions during pregnancy is warranted. These patients should be treated similarly to their non-gravid counterparts. There are also several additional risk factors that increase the likelihood of AVM hemorrhage and may be considered when indications for surgery are not clear. These include hypertension, increased age, coagulopathy, disseminated intravascular coagulation and recent use of vasoactive substances [11]. Of these risk factors, our patient had untreated gestational hypertension, which is the single factor most closely linked with AVM rupture [11].

Endovascular embolization has been used with increasing frequency for the treatment of AVMs in the general population. Embolization may either be performed as stand-alone treatment or as part of a multimodal therapy. When embolization is used alone for the definitive treatment of AVMs, the reported cure rates are 5% to 28%, lower than the cure rates of surgical resection (80% to 95%) and radiosurgery (65% to 85%). In addition, stand-alone embolic therapy has relatively high morbidity (4% to 9%) and mortality (2% to 4%) when compared to surgical resection and radiosurgery. These complications are due, largely, to the fact that the more complicated AVMs tend to be the ones that receive endovascular treatment [7,9,12]. The utility of endovascular embolization, however, becomes more apparent when used prior to surgical resection. In such cases the embolization typically reduces the size of the AVM and may improve safety during the subsequent resection. For instance, it is thought that reducing the AVM in a step-wise fashion with embolization lowers the risks for normal perfusion pressure break-through syndrome, where a diversion of the AVM's blood towards other vessels leads to cerebral edema and hemorrhage [8]. Also, because embolization during multimodal therapy is less aggressive than during stand-alone therapy, the morbidity (4% to 6%) and mortality (0% to 2%) tend to be lower [8,13,14]. There is a lack of studies that compare cure rates and complications of endovascular embolization with surgery versus surgery alone. In a retrospective study, Deruty *et al. *[15] had an 82% cure rate in 19 patients managed with surgery alone and a 100% cure rate in 19 patients managed with both embolization and surgery, suggesting a benefit to embolization before surgery.

For small AVMs or lesions in easily accessible areas, surgery alone may be suitable. However, when lesions are large, in deep areas or have many feeders, embolization before surgery may increase the cure rate while reducing the overall risk. When these procedures are performed in pregnant women, proper shielding of the abdomen and minimizing the use of ionizing radiation are of particular importance. The use of selective angiography, for example, minimizes fluoroscopy time and radiation exposure. Visipaque™ is not contraindicated in pregnancy; however, use of half strength contrast may further increase safety. In addition, while Onyx® embolization was effective in this case, an argument could be put forth for using N-butyl 2-cyanoacrylate, for which the actual embolization process is considerably quicker. The involvement of a high-risk obstetric team with fetal heart monitoring capability also provides valuable protection. Experience with intravascular procedures in pregnant women will further help guide management of this rare, but important patient population.

## Conclusions

The rupture of an AVM in a pregnant patient is a serious complication that requires surgical intervention. The use of endovascular embolization with surgical resection is safe in these patients, particularly when measures are taken to protect the fetus, and may provide benefits over the use of surgery or embolization alone.

## Consent

Written informed consent was obtained from the patient for publication of this case report and any accompanying images. A copy of the written consent is available for review by the Editor-in-Chief of this journal.

## Competing interests

The authors declare that they have no competing interests.

## Authors' contributions

WJJ prepared the majority of the manuscript. LDT and MG also contributed significantly to the manuscript and participated in the surgical procedures. RJS was the attending surgeon. All authors read and approved the final manuscript.
